# Gene tree and species tree reconciliation with endosymbiotic gene transfer

**DOI:** 10.1093/bioinformatics/btab328

**Published:** 2021-07-12

**Authors:** Yoann Anselmetti, Nadia El-Mabrouk, Manuel Lafond, Aïda Ouangraoua

**Affiliations:** 1 Département d'informatique, Université de Sherbrooke, 2500, boulevard de l'Université, Sherbrooke (Québec) J1K 2R1, Canada; 2 Département d'informatique et de recherche opérationnelle, Université de Montréal, CP 6128 succ Centre-Ville, Montréal, Québec H3C 3J7, Canada

## Abstract

**Motivation:**

It is largely established that all extant mitochondria originated from a unique endosymbiotic event integrating an α−proteobacterial genome into an eukaryotic cell. Subsequently, eukaryote evolution has been marked by episodes of gene transfer, mainly from the mitochondria to the nucleus, resulting in a significant reduction of the mitochondrial genome, eventually completely disappearing in some lineages. However, in other lineages such as in land plants, a high variability in gene repertoire distribution, including genes encoded in both the nuclear and mitochondrial genome, is an indication of an ongoing process of Endosymbiotic Gene Transfer (EGT). Understanding how both nuclear and mitochondrial genomes have been shaped by gene loss, duplication and transfer is expected to shed light on a number of open questions regarding the evolution of eukaryotes, including rooting of the eukaryotic tree.

**Results:**

We address the problem of inferring the evolution of a gene family through duplication, loss and EGT events, the latter considered as a special case of horizontal gene transfer occurring between the mitochondrial and nuclear genomes of the same species (in one direction or the other). We consider both EGT events resulting in maintaining (EGTcopy) or removing (EGTcut) the gene copy in the source genome. We present a linear-time algorithm for computing the DLE (Duplication, Loss and EGT) distance, as well as an optimal reconciled tree, for the unitary cost, and a dynamic programming algorithm allowing to output all optimal reconciliations for an arbitrary cost of operations. We illustrate the application of our EndoRex software and analyze different costs settings parameters on a plant dataset and discuss the resulting reconciled trees.

**Availability and implementation:**

EndoRex implementation and supporting data are available on the GitHub repository via https://github.com/AEVO-lab/EndoRex.

## 1 Introduction

Genomics and cell biology investigations have revealed that all known eukaryotes descend from a common ancestral mitochondrial-containing cell that originated from the integration of an endosymbiotic α-proteobacterium into a host cell (Dyall and Johnson, 2000). After this early event, eukaryotic gene contents have been shaped by duplications, losses and Horizontal Gene Transfers (HGT) from one species to another, but also by Endosymbiotic Gene Transfers (EGT), mainly from the mitochondrion to the nucleus, in some cases leading to the total disappearance of the mitochondrion (Roger et al., 2017; Sloan et al., 2018).

Many questions regarding the ancestral mitochondrial proteome and gene content evolution remain open (Lang and Burger, 2012). One of the reasons is that, to date, comparative genomics studies have largely focused on multicellular eukaryotes, mainly animals and plants. While imprints of global evolutionary events at the genomic level are hardly visible on multicellular eukaryotes that have diverged too much from the Last Eukaryotic Common Ancestor (LECA), protists, known to have emerged close to the eukaryotic origin, are better candidates for such a comprehensive evolutionary study. Interestingly, a recent sequencing effort on jakobids (Gray et al., 2020) and malawimonads (Derelle et al., 2015) protist genomes have been undertaken by a consortium of protistologists (DeepEuk), suggesting that soon enough data will be available to allow further investigations on early-eukaryotic evolution.

In addition to having the appropriate datasets, understanding the concerted evolution of the eukaryotic mitochondrial and nuclear genomes also requires having the appropriate algorithmic tools. This problem can be seen as related to the host-parasite coevolution inference problem (Charleston and Perkins, 2006). Given a host tree and a parasite tree, cophylogenetic analysis consists in inferring a history of codivergence, parasite duplication, host switch or extinction events explaining the coevolution of hosts and parasites. However, nuclear and mitochondrial genomes can hardly be treated by the same kind of approach, as they evolve, through a different evolutionary model, together in the same species, and thus are related through the same species tree. Rather, inferring an endosymbiotic evolutionary history requires focusing on gene families and studying the movement of genes between the mitochondrial and nuclear genomes.

Inferring the evolution of gene families is the purpose of the gene-tree-species-tree-reconciliation field, seeking for a most parsimonious (El-Mabrouk and Noutahi, 2019; Goodman et al., 1979), or a most probable (Akerborg et al., 2009; Szöllősi et al., 2015) evolutionary scenario of gene gain and loss explaining the incongruence between a gene tree and a species tree. A most parsimonious reconciliation minimizing the number of Duplications (the D-distance) or the number of Duplications and Losses (the DL-distance) can be found in linear time using the LCA (Last Common Ancestor) mapping (Chen, 2000; Zhang, 1997; Zmasek and Eddy, 2001). Such an algorithm can actually be used to solve the cophylogenetic problem if operations are restricted to coevolution, duplication and extinction. Including HGT events (i.e. finding the DTL-distance) leads to an NP-hard problem if time-consistency is required, remaining polynomial otherwise (Bansal et al., 2012; Tofigh et al., 2011).

In this article, we introduce the reconciliation model accounting for EGT events, i.e. the special case of HGT events where genes are exchanged only between the mitochondrial and nuclear genomes of the same species. Although integration of the mitochondrial content into the nucleus is the most frequent event in the course of evolution of eukaryotes, the transfer from the nucleus to the mitochondrion has also been observed (Adams and Palmer, 2003). Here, we consider the exchange of genes in both directions. Moreover, we consider EGT events resulting in maintaining a gene copy in the source genome (EGTcopy), as well as those resulting in the removal or loss of function of the gene in the source genome (EGTcut).

Formally, given a gene tree for a gene family with a known mitochondrial or nuclear location for each gene copy, we seek for a most parsimonious sequence of Duplication, Loss and EGT (DLE) events explaining the tree given a known species tree. First, based on the DL-distance and on the Fitch algorithm for weighted parsimony, we present, in Section 3, a linear-time algorithm for computing the DLE-Distance, as well as an optimal reconciled tree for the unitary cost. We then develop, in Section 4, a general dynamic programming algorithm that can be used to output all optimal reconciliations, for an arbitrary cost of operations, including possibly a different cost for an EGT from the mitochondrion to the nucleus, or conversely. This algorithm is linear in the size of the gene tree. It can be seen as an adaptation of the quadratic-time DTL algorithm for dated trees (Doyon et al., 2010), which allows transfers between any co-existing species. We finally illustrate, in Section 5, the application of our EndoRex software on clusters of orthologous mitochondrial protein-coding genes (MitoCOGs) (Kannan et al., 2014) of plants, analyze different costs settings parameters and discuss the obtained reconciled trees.

For space reasons, some of the proofs are given in Appendix.

## 2 Preliminaries

All trees are considered rooted. Given a tree *T*, we denote by *r*(*T*) its root, by *V*(*T*) its set of nodes and by ℓ(T)⊆V(T) its leafset. A node *x* is a *descendant* of x′ if *x* is on the path from x′ to a leaf of *T* and an *ancestor* of x′ if *x* is on the path from *r*(*T*) to x′;*x* is a *strict descendant* (respectively *strict ancestor*) of x′ if it is a descendant (respectively ancestor) of x′ different from x′. Moreover, *x* is the *parent* of x′≠r(T) if it directly precedes x′ on the path from x′ to *r*(*T*). In this latter case, x′ is a *child* of *x*. We denote by *E*(*T*) the set of edges of *T*, where an edge is represented by its two terminal nodes (x,x′), with *x* being the parent of x′. An internal node (a node which is not a leaf) is said to be *unary* if it has a single child and *binary* if it has two children. If not stated differently, the children of a binary node *x* are denoted *x_l_* and *x_r_*. Given a node *x* of *T*, the subtree of *T* rooted at *x* is denoted T[x].

A *binary tree* is a tree with all internal nodes being binary. If internal nodes have one or two children, then the tree is said *partially binary*.

The *lowest common ancestor* (LCA) in *T* of a subset L′ of ℓ(T), denoted $lca_{T}(L\prime )$, is the ancestor common to all the nodes in L′ that is the most distant from the root.

A tree *R* is *an extension* of a tree *T* if it is obtained from *T* by *grafting* unary or binary nodes in *T*, where grafting a unary node *x* on an edge (*u*, *v*) consists in creating a new node *x*, removing the edge (*u*, *v*) and creating two edges (*u*, *x*) and (*x*, *v*), and in the case of grafting a binary node, also creating a new leaf *y* and an edge (*x*, *y*). In the latter case, we say that *y* is a grafted leaf.

*Species and gene trees:* The *species tree S* for a set Σ of species represents a partially ordered set of speciation events that have led to Σ. In this article, we consider that each species of σ∈Σ has two genomes: **_*σ*_**_0_ corresponding to its mitochondrial genome and **_*σ*_**_1_ corresponding to its nuclear genome.

A *gene family* is a set Γ of genes where each gene *x* belongs to a given species *s*(*x*) of Σ. A tree *T* is a *gene tree* for a gene family Γ if its leafset is in bijection with Γ. We will make no distinction between a leaf of *T* and the gene of _**Γ**_ it corresponds to. We call *s*(*x*) the *species labeling* of the leaf *x*. For a subset G⊆Γ of genes, we write s(G)={s(g):g∈G} as the set of species containing the genes of *G*.

Moreover, we assign to each gene *x* of Γ a Boolean value corresponding to the genome it belongs to. More precisely, *b*(*x*) = 0 if *x* belongs to $s{\left(x\right)}_{0}$ and *b*(*x*) = 1 if *x* belongs to $s{\left(x\right)}_{1}$. In this article, we assume that the mitochondrial or nuclear location of each extant gene is known. We call *b*(*x*) the *genome labeling* of the leaf representing *x*.

An evolutionary history is represented by an *event labeled* tree, where the event label $\tilde{e}(x)$ of an internal node *x* is its corresponding event. The event labeling of the internal nodes of a gene tree is obtained through reconciliation.

### 2.1 Reconciliation

**Fig. 1.**
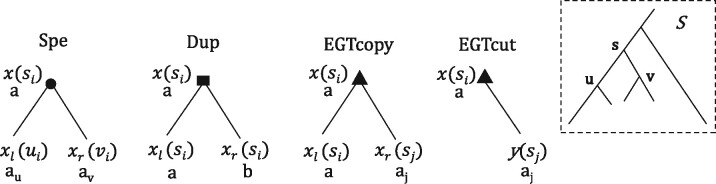
The effect of an event on a node *x* of a gene tree representing the gene *a* belonging to the genome *s_i_* (denoted $x(s_{i})$), where *s* is a species and i∈{0,1} (for a species *s*, *s_o_* is the mitochondrial genome and *s*_1_ the nuclear genome of *s*). The tree *S* up-right is the species tree, where *u* and *v* are the two species arising from the speciation of *s*. (Spe): Gives rise to a copy *a_u_* in *u_i_* and *a_v_* in *v_i_*; (Dup): Preserves the copy *a* in *s_i_* and gives rise to a new copy *b* in *s_i_*; (EGTcopy): Represents a transfer event from *s_i_* to *s_j_*, where j∈{0,1} and j≠i, preserving the copy *a* in *s_i_* and giving rise to a new copy *a_j_* in *s_j_*; (EGTcut): Represents a transposition event from *s_i_* to *s_j_* removing the copy *a* in *s_i_* and creating a copy *a_j_* in *s_j_*

Inside the species’ genomes, genes undergo *Speciation* (Spe) when the species to which they belong do, but also *Duplication* (Dup) i.e. the creation of a new gene copy, *Loss* of a gene copy and *Horizontal Gene Transfer* (HGT) when a gene is transmitted from a source to a target genome. In this article, we consider special cases of HGTs, called EGTs, only allowing the transmission of genes from the mitochondrial genome to the nuclear genome of the same species, or vice-versa. Moreover, we consider two types of EGTs: *EGTcopy* and *EGTcut* defined as follows (see Fig. 1):

- A gene *x* belonging to **_*σ*_**_*i*_ is *copied* (or transferred) by an EGTcopy event to **_*σ*_**_*j*_ for {i,j}={0,1} if it is copied from **_*σ*_**_*i*_ and inserted in **_*σ*_**_*j*_.
- A gene *x* belonging to **_*σ*_**_*i*_ is *transposed* by an EGTcut event to **_*σ*_**_*j*_ for {i,j}={0,1} if it is cut from **_*σ*_**_*i*_ and inserted in **_*σ*_**_*j*_.

Thus, in this article, the set of considered events is:

DLE={Spe,Dup,Loss,EGTcopy,EGTcut}

Notice that we do not consider general HGT events. To define a DLE-Reconciliation, assume that we are given a species tree *S*, a gene tree *T*, a mapping *s* from ℓ(T) to ℓ(S) and a mapping *b* from ℓ(T) to {0, 1}. We need to define how to extend *s* and *b* to the internal nodes of *T*. Given an extension *R* of *T* (*R* can be equal to *T*) *an extension of s* is a function $\tilde{s}$ from *V*(*R*) to *V*(*S*) such that, for each leaf *x* of *T*, $\tilde{s}(x)=s(x)$. Moreover, an *extension of b* is a function $\tilde{b}$ from *V*(*R*) to {0, 1} such that, for each leaf *x* of *T*, $\tilde{b}(x)=b(x)$.

Definition 1 (DLE-Reconciliation). *Let* **_*Γ*_** *be a gene family where each* x∈Γ*belongs to the genome b(x) of a species s(x) of* **_*Σ*_***. Let T be a rooted binary gene tree for* **_*Γ*_** *and S be a rooted binary species tree for* **_*Σ*_***. A DLE-Reconciliation is a quadruplet* $\langle R,\tilde{s},\tilde{b},\tilde{e}\rangle$*where R is a partially binary extension of T*, $\tilde{s}$*is an extension of s and* $\tilde{b}$*is an extension of b such that:*

a. $\tilde{s}(x_{l})$*and* $\tilde{s}(x_{r})$*are the two children of* $\tilde{s}(x)$*in S and* $\tilde{b}(x_{l})=\tilde{b}(x_{r})=\tilde{b}(x)$*, in which case* $\tilde{e}(x)=\mathrm{Spe};$
b. $\tilde{s}(x_{l})=\tilde{s}(x_{r})=\tilde{s}(x)=\sigma$*and* $\tilde{b}(x_{l})=\tilde{b}(x_{r})=\tilde{b}(x)$*in which case* $\tilde{e}(x)=\mathrm{Dup}$*representing a duplication in* $\sigma_{\tilde{b}(x)};$
c. $\tilde{s}(x_{l})=\tilde{s}(x_{r})=\tilde{s}(x)=\sigma$*and* $\tilde{b}(x_{l})\neq \tilde{b}(x_{r})$*in which case* $\tilde{e}(x)=\mathrm{EGTcopy};$*let y be the element of* $\left\{x_{l},x_{r}\right\}$*such that* $\tilde{b}(x)\neq \tilde{b}(y)$*, then* $\tilde{e}(x)$*is a transfer with source genome* $\sigma_{\tilde{b}(x)}$*and target genome* $\sigma_{\tilde{b}(y)}$.

*A grafted leaf on a newly created node x corresponds to a loss in* $\tilde{s}(x)$.

As *R* is as an extension of *T*, each node in *T* has a corresponding node in *R*. In other words, we can consider that V(T)⊆V(R). In particular, the species labeling on *R* induces a species labeling on *T*.

Given a cost function *c* on *DLE* and a reconciliation $R=\langle R,\tilde{s},\tilde{b},\tilde{e}\rangle$, the cost c(R) is the sum of costs of the induced events. In this article, we assume a 0 cost for speciations and positive costs for all the other events.

We are now ready to formally define the considered optimization problem.

**DLE-Reconciliation Problem:**

**Input:** A species tree *S* for a set of species Σ, a gene family Γ on Σ, a gene tree *T* for Γ, a species labeling *s* and a genome labeling *b* of ℓ(T), and a cost function *c* on DLE.

**Output:** A most *parsimonious DLE-Reconciliation*, i.e. a DLE-Reconciliation $\langle R,\tilde{s},\tilde{b},\tilde{e}\rangle$ of minimum cost.

In the next section, we first consider the case of a unitary cost, thus reducing the problem to minimizing the number of operations induced by a reconciliation. The cost *DLE*(*T*, *S*) of the most parsimonious DLE-Reconciliation for *T* and *S* in the case of a unitary cost *c* is called the *DLE-Distance*. We then extend the algorithmic developments to arbitrary costs, allowing in particular to consider an EGTcopy or an EGTcut event copying a gene from the mitochondria to the nucleus differently from a similar event copying a gene from the nucleus to the mitochondria.

In the following section, we will refer to the DL-Reconciliation of *T* and *S*. Recall that it is a triplet $\langle R_{DL},\tilde{s},\tilde{e}\rangle$ defined by only considering the cases of speciations, duplications and losses in Definition 1, and ignoring the binary assignment of genes. We denote by *DL*(*T*, *S*) the DL-Distance, i.e. the minimum number of duplications and losses induced by a DL-reconciliation. The DL-Reconciliation $\langle R_{DL},\tilde{s},\tilde{e}\rangle$ of cost *DL*(*T*, *S*) is unique and verifies, for any internal node *x* of $V(R_{DL})\cap V(T)$:

1.
$\tilde{s}(x)=lca_{S}(s(\ell (T[x])));$

2. if $\tilde{s}(x)\neq \tilde{s}(x_{l})$ and $\tilde{s}(x)\neq \tilde{s}(x_{r})$ then *v* is a Speciation; otherwise *x* is a Duplication.

We finally need to make the link between the species labeling $\tilde{s}$ of an optimal reconciliation and the well-known LCA-Mapping. This is formally stated in the following lemma.

Lemma 1 (LCA-Mapping). *Let* $\langle R,\tilde{s},\tilde{b},\tilde{e}\rangle$*be a DLE-Reconciliation of minimum cost between T and S. Then, for each* $x\in V(T)\cap V(R),\;\tilde{s}(x)=lca_{S}(s(\ell (T[x])))$.

Note that in the above statement, V(T)∩V(R)=V(T), and thus the intersection is redundant. We write it this way to emphasize that *x* is a vertex of *R* (which happens to also be in *T*), i.e. the LCA-Mapping here applies to the reconciled trees, not to the original gene tree *T*.

## 3 A linear-time algorithm for the DLE-distance

In this section, we consider a unitary cost *c* on DLE.

Consider a *given* extension ${\tilde{b}}_{T}$ of *b* to the internal nodes of *T*. We first present an algorithm for computing a DLE-Reconciliation $\langle R,\tilde{s},\tilde{b},\tilde{e}\rangle$ of minimum cost, under the condition that $\tilde{b}(x)={\tilde{b}}_{T}(x)$ for each x∈V(T)∩V(R). We will then show how a ${\tilde{b}}_{T}$ minimizing the DLE-Distance can be obtained.

**Fig. 2.**
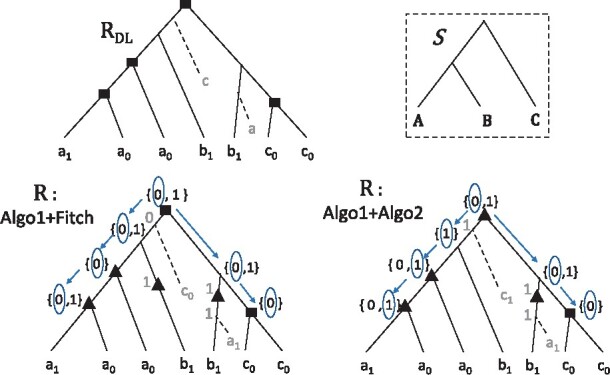
The tree *R_DL_* up left, together with its node labeling, is the optimal DL-Reconciliation for the gene tree *T* represented by the plain edges of *R_DL_* and the species tree *S* up right. The two down trees are obtained by Algorithm 1 for two different $\tilde{b}$ labeling of internal nodes: the left labeling is obtained by the Fitch algorithm for weighted parsimony, while the right labeling is obtained by applying Algorithm 2. The left labeling gives rise to a non-optimal reconciliation with seven operations (two losses, one duplication, two EGTcopy and two EGTcut), while the right labeling gives rise to the DLE-Distance which is equal to six (two losses, three EGTcopy and one EGTcut). Rectangles represent duplications; triangles represent either EGTcopy or EGTcut events depending whether the labeled node is binary or unary; dotted lines represent losses; A leaf *x_i_* represent a gene *x* belonging to the genome *i* (0 for mitochondrial and 1 for nuclear) of species *X*

Algorithm 1 computes the DLE-Reconciliation $\langle R,\tilde{s},\tilde{b},\tilde{e}\rangle$ from the DL-Reconciliation $\langle R_{DL},{\tilde{s}}_{DL},{\tilde{e}}_{DL}\rangle$ (see Fig. 2 for an example).

Lemma 2 (Optimality of Algorithm 1). *Given a binary assignment* ${\tilde{b}}_{T}$*of the nodes of T, Algorithm 1 outputs a DLE-Reconciliation* $\langle R,\tilde{s},\tilde{b},\tilde{e}\rangle$*of minimum cost with the constraint that* $\tilde{b}(x)={\tilde{b}}_{T}(x)$*for* x∈V(R)∩V(T).

It follows from Lemma 2 that if $\tilde{b}$ is known in advance for the nodes of *T*, a DLE-Reconciliation of minimum cost is obtained from Algorithm 1 with $\tilde{b}$ as input. We now focus on finding such a labeling $\tilde{b}$.

Lemma 3 (Necessary condition for $\tilde{b}$) *There exists a DLE-Reconciliation* $\langle R,\tilde{s},\tilde{b},\tilde{e}\rangle$*of minimum cost DLE(T, S) such that, for any node x of T and its children x_l_ and x_r_ in T*, $\tilde{b}(x)=\tilde{b}(x_{l})$*or* $\tilde{b}(x)=\tilde{b}(x_{r})$.

Proof.

Assume $\langle R,\tilde{s},\tilde{b},\tilde{e}\rangle$ is a most parsimonious DLE-Reconciliation with a lowest node *x* not satisfying condition (1): $\tilde{b}(x)=\tilde{b}(x_{l})$ or $\tilde{b}(x)=\tilde{b}(x_{r})$. Thus we should have $\tilde{b}(x)\neq \tilde{b}(x_{l})=\tilde{b}(x_{r})$. Note that an EGTcut event must be present on at least one of the $\left(x,x_{l}\right)$ or $\left(x,x_{r}\right)$ branches. A reconciliation of lower or equal cost can be obtained by assigning $\tilde{b}(x)=\tilde{b}(x_{l})=\tilde{b}(x_{r})$ and removing this EGTcut event, reducing the cost by one. Let *p_x_* be the parent of *x* in *R* (note that if *x* is the root, *p_x_* might not exist, in which case there is nothing else to do). If $\tilde{b}(x)$ is now different from $\tilde{b}(p_{x})$, we add an EGTcut event between *p_x_* and *x*, yielding an alternate reconciliation of equal or lower cost.

We can reproduce the same transformation iteratively in a bottom-up fashion until condition (1) is satisfied for every node. _**□**_

For a node x∈V(T), define *d*(*x*) = 1 if *x* is a duplication in the DL-Reconciliation of minimum cost, and *d*(*x*) = 0 otherwise. Let $\tilde{b}$ be a binary labeling of *V*(*T*). For any node *x* of *T*, denote $\Delta_{\tilde{b}}(x)=0$ if x∈ℓ(T), otherwis*e*

$$\Delta_{\tilde{b}}(x)=\max(0,|\tilde{b}(x)-\tilde{b}(x_{l})|+|\tilde{b}(x)-\tilde{b}(x_{r})|-d(x))$$

and define:

$$\mathrm{cost}(T,S,\tilde{b})=\sum_{x\in V(T)}\Delta_{\tilde{b}}(x)$$

Roughly speaking, $\Delta_{\tilde{b}}(x)$ reflects the number of label changes between *x* and its children *x_l_* and *x_r_* in *T*, with the exception that a duplication is allowed a ‘free’ change since it can be turned into an EGTcopy node. For example, in Figure 2, $\mathrm{cost}(T,S,\tilde{b})=2$ for the labeling $\tilde{b}$ of *T* consistent with that of the left tree *R* (Algo1+Fitch), and $\mathrm{cost}(T,S,\tilde{b})=1$ for the labeling $\tilde{b}$ of *T* consistent with that of the right tree *R* (Algo1+Algo2), reflecting, for each one, the number of requested EGTcut.

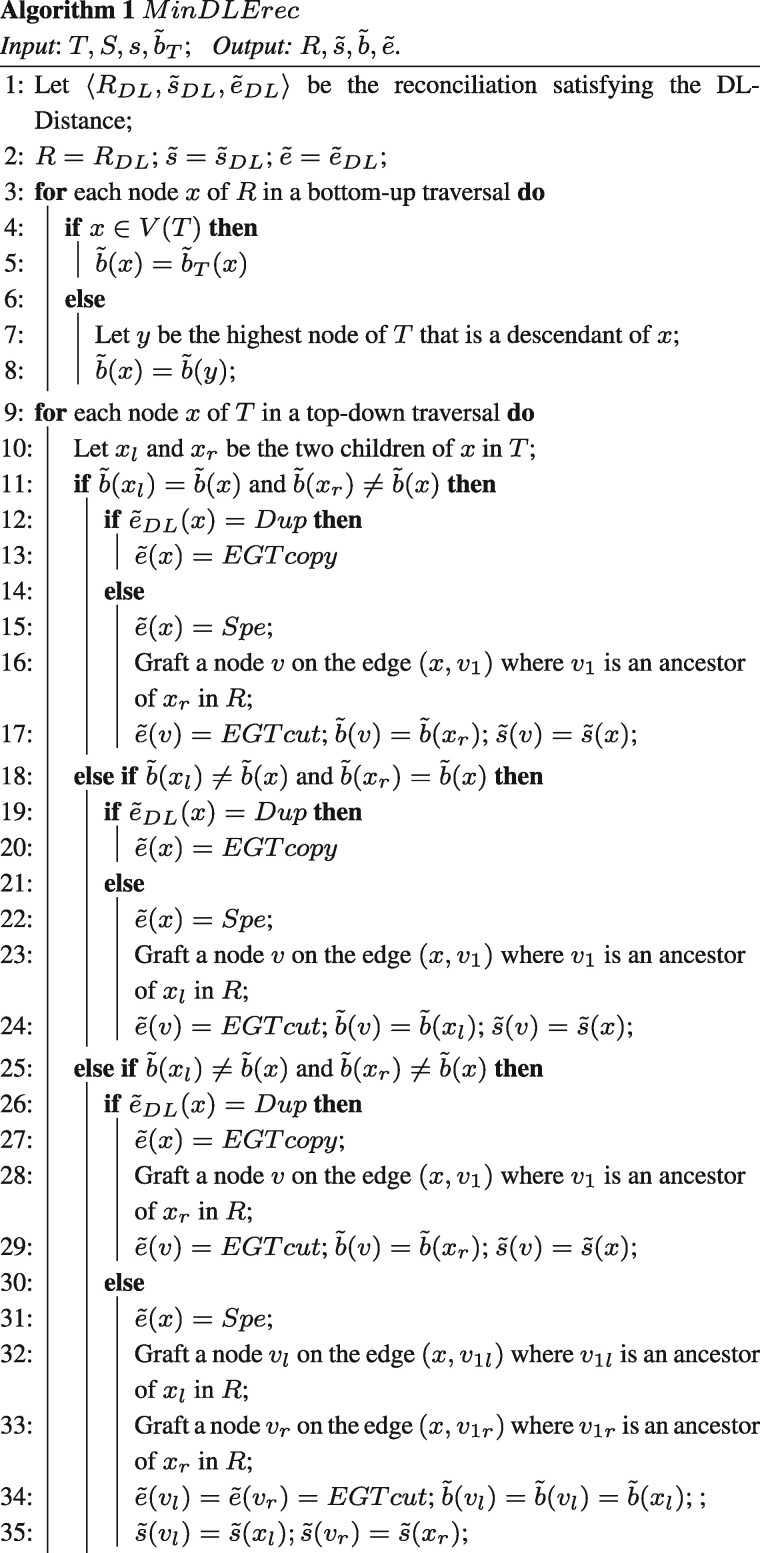

Lemma 4. *The minimum cost of a DLE-Reconciliation between a gene tree T and a species tree S is*

$$\mathrm{DLE}(T,S)=DL(T,S)+\min_{\tilde{b}}c\mathrm{ost}(T,S,\tilde{b})$$

Proof. By Lemma 2, Algorithm 1 correctly infers a minimum cost DLE-Reconciliation for a given $\tilde{b}$. Note that this DLE-Reconciliation is obtained from a DL-Reconciliation by turning some duplication nodes into EGTcopy nodes (which do not change the cost), and by grafting some EGTcut nodes. Thus, the latter are responsible for any possible change in cost from *DL*(*T*, *S*) to *DLE*(*T*, *S*). It follows that the cost of the returned DLE-Reconciliation is *DL*(*T*, *S*), plus the number of grafted EGTcut nodes.

Let $\tilde{b}$ be a binary assignment of *T* that minimizes *DLE*(*T*, *S*) when $\tilde{b}$ is passed to Algorithm 1. By Lemma 3, we may assume that for any node *x* and its children *x_l_* and *x_r_*, $\tilde{b}(x)=\tilde{b}(x_{l})$ or $\tilde{b}(x)=\tilde{b}(x_{r})$. Thus $\Delta_{\tilde{b}}(x)\in \{0,1\}$ for every *x*. Furthermore, $\Delta_{\tilde{b}}(x)=1$ if and only if *x* is a speciation node and an EGTcut node is grafted on the edge $\left(x,x_{l}\right)$ (if $\tilde{b}(x)\neq \tilde{b}(x_{l})$) or on the edge $\left(x,x_{r}\right)$ (if $\tilde{b}(x)\neq \tilde{b}(x_{r})$). In consequence, $\mathrm{cost}(T,S,\tilde{b})$ counts exactly the number of graftings of EGTcut nodes. _**□ □**_

Since the most-parsimonious DL-Reconciliation is unique, the *DL*(*T*, *S*) term in the above lemma is an invariant. Our goal is therefore to find the labeling $\tilde{b}$ that minimizes $\mathrm{cost}(T,S,\tilde{b})$.

This can be achieved by a slight modification of the Fitch (1971) algorithm (Fitch, 1971) computing, for a given tree with leaf labels, all possible label assignments of internal nodes minimizing the number of label changes along the edges of the tree. We first need to recall some concepts on parsimony. Given a tree *T* on a leafset *L* of residues (generally nucleotides or amino-acids, but in this article L={0,1} corresponding to the possible $\tilde{b}$ labeling), the *weighted parsimony* problem consists in assigning a residue $\tilde{b}(u)\in L$ to each internal node *u* of *T* in a way minimizing the total weight of the tree. More precisely, given a cost matrix *M* on residues, the weight of *T* is the sum of weights $M(\tilde{b}(u),\tilde{b}(v))$ for all (u,v)∈E(T). An *assignment of T* refers to the assignment of a residue to each internal node of *T*.

The Sankoff and Cedergren (1983) algorithm (Sankoff and Cedergren, 1983) allows to compute, in quadratic time, the minimum cost min(T) of an assignment of *T*. Moreover, it allows to find all the assignments $\tilde{T}$ of *T* leading to min(T). When M(a,a)=0 for all a∈L and M(a,b)=1 for a≠b, weighted parsimony can be computed in linear time using the Fitch algorithm.

The Fitch algorithm consists of two phases. The first phase is recursive and reconstructs possible ancestral labels *L*(*x*) for each node *x* of *T* and the overall minimum number of label changes required as follows: For each node *x* of *T* in a bottom-up traversal, (1) if *x* is a leaf, then $L(x)=\{\tilde{b}(x)\}$ and cost(T[x])=0. (2) Else, let *x_l_* and *x_r_* be the children of *x*. If $L(x_{l})\cap L(x_{r})=\emptyset$, then $L(x)=L(x_{l})\cup L(x_{r})$ and $\mathrm{cost}(T[x])=\mathrm{cost}(T[x_{l}])+\mathrm{cost}(T[x_{r}])+1$; else $L(x)=L(x_{l})\cap L(x_{r})$ and $\mathrm{cost}(T[x])=\mathrm{cost}(T[x_{l}])+\mathrm{cost}(T[x_{r}])$. The second phase of the algorithm reconstructs an assignment $\tilde{b}$ of *T* that has a minimum cost, by computing $\tilde{b}(x)$ as follows: For each node *x* of *T* in a top-down traversal, (1) if *x* is the root, assign $\tilde{b}(x)$ to any label in *L*(*x*). (2) Else, let *x_p_* be the parent of *x*. If $\tilde{b}(x_{p})\in L(x)$, then assign $\tilde{b}(x)=\tilde{b}(x_{p})$, else assign $\tilde{b}(x)$ to any label in *L*(*x*).

The Fitch algorithm does not always find an optimal $\tilde{b}$ assignment because of duplications that can be turned into EGTcopy events. Algorithm 2 modifies the first phase of the Fitch algorithm to compute the DLE-Distance and an assignment $\tilde{b}$ of *T* that leads to the DLE-Distance. The modification reflects the fact that a duplication node is allowed a ‘free’ change since it can be turned into an EGTcopy node (see Fig. 2 for an illustration).

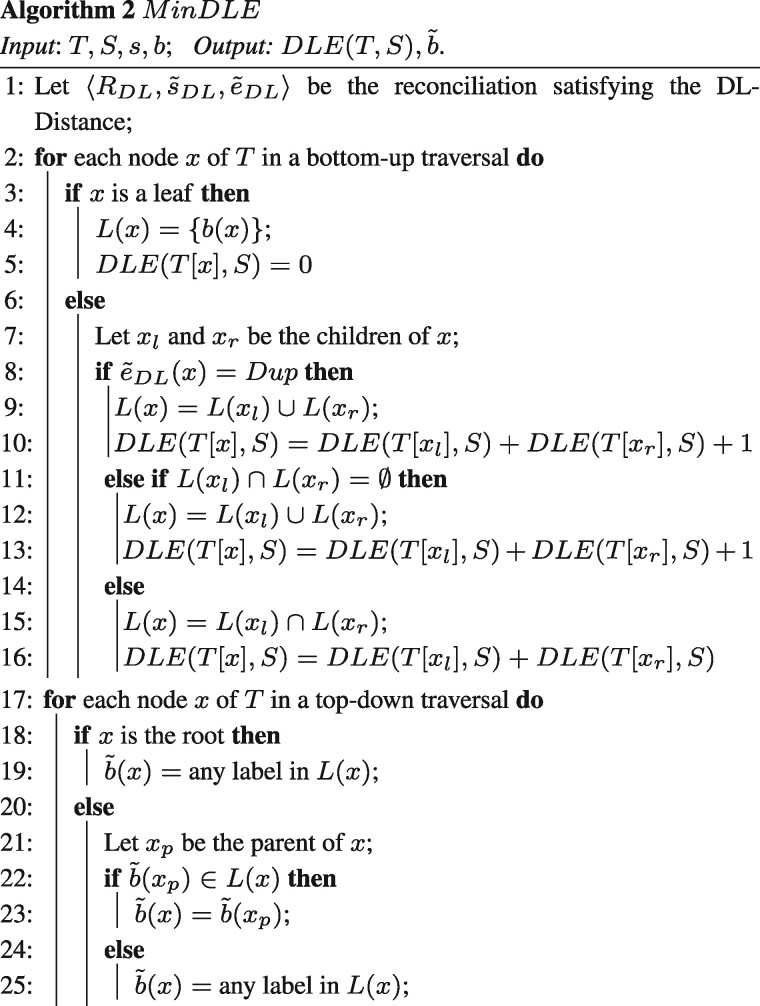

Lemma 5. *Algorithm 2 outputs, in linear time, the DLE-Distance DLE(T, S) and a binary assignment* $\tilde{b}$*of T that leads to a most parsimonious DLE-Reconciliation.*

Proof. It suffices to prove that the following statement holds for any node *x* of *T*: for any label **_*β*_** in *L*(*x*), there exists a binary assignment $\tilde{b}$ of T[x] such that $\tilde{b}(x)=\beta$ and $\tilde{b}$ minimizes $\mathrm{cost}(T[x],S,\tilde{b})$.

1. If $\beta \in L(x_{l})\cap L(x_{r})$, then $\tilde{b}(x_{l})=\tilde{b}(x_{r})=\tilde{b}(x)=\beta$, and $\Delta_{\tilde{b}}(x)=0$. Thus $\mathrm{cost}(T[x],S,\tilde{b})=\mathrm{cost}(T[x_{l}],S,{\tilde{b}}_{l})+\mathrm{cost}(T[x_{r}],S,{\tilde{b}}_{r})$, without any increment.
2. If β∈L(xl)∩L(xr), then $\beta \in L(x_{l})$ or $\beta \in L(x_{r})$, and $\tilde{b}(x_{l})=\tilde{b}(x)=\beta$ or $\tilde{b}(x_{r})=\tilde{b}(x)=\beta$, and $\Delta_{\tilde{b}}(x)=0$. Thus $\mathrm{cost}(T[x],S,\tilde{b})=\mathrm{cost}(T[x_{l}],S,{\tilde{b}}_{l})+\mathrm{cost}(T[x_{r}],S,{\tilde{b}}_{r})$, without any increment.
  In both cases, Algorithm 1 computes a DLE-Reconciliation with minimum cost $\mathrm{DLE}(T[x_{l}],S)+\mathrm{DLE}(T[x_{r}],S)+1$ with a minimum increment of 1 for a Dup node in case (1), or by making *x* an EGTcopy node in case (2), but no additional EGTcut node is required.
3. If *x* is a speciation node in the DL-reconciliation.

1. If $L(x)\neq L(x_{l})\cap L(x_{r})$, then $L(x_{l})\cap L(x_{r})=\emptyset$, and $\beta \in L(x_{l})$ or $\beta \in L(x_{r})$. So $\tilde{b}(x_{l})=\tilde{b}(x)=\beta$ or $\tilde{b}(x_{r})=\tilde{b}(x)=\beta$, and $\Delta_{\tilde{b}}(x)=1$. Thus $\mathrm{cost}(T[x],S,\tilde{b})=\mathrm{cost}(T[x_{l}],S,{\tilde{b}}_{l})+\mathrm{cost}(T[x_{r}],S,{\tilde{b}}_{r})+1$, with a minimum increment of 1, obtained by grafting an EGTcut node on one of the $\left(x,x_{l}\right)$ or $\left(x,x_{r}\right)$ branches. In this case, Algorithm 1 computes a DLE-Reconciliation with minimum cost $\mathrm{DLE}(T[x_{l}],S)+\mathrm{DLE}(T[x_{r}],S)+1$.
2. If $L(x)=L(x_{l})\cap L(x_{r})$, then $\beta \in L(x_{l})$ and $\beta \in L(x_{r})$. So $\tilde{b}(x_{l})=\tilde{b}(x_{r})=\tilde{b}(x)=\beta$, and $\Delta_{\tilde{b}}(x)=0$. Thus $\mathrm{cost}(T[x],S,\tilde{b})=\mathrm{cost}(T[x_{l}],S,{\tilde{b}}_{l})+\mathrm{cost}(T[x_{r}],S,{\tilde{b}}_{r})$ without any additional cost. Algorithm 1 computes a DLE-Reconciliation with minimum cost $\mathrm{DLE}(T[x_{l}],S)+\mathrm{DLE}(T[x_{r}],S)$ when given $\tilde{b}$.

It is easy to see that both the first and the second phases of the algorithm have linear time complexity, thus the overall algorithm has a linear time complexity. _**□**_

As for the Fitch Algorithm, Algorithm 2 does not allow to output all the solutions of the DLE-Reconciliation problem leading to the DLE-Distance. However, this can be achieved by adapting the Sankoff and Cedergren’s dynamic programming algorithm. Rather, we choose to introduce, in the next section, a more general dynamic programming algorithm allowing to output all optimal solutions for an arbitrary cost of the DLE events, not only for the unitary cost.

## 4 Solving the DLE-reconciliation problem with arbitrary DLE costs

We now introduce a dynamic programming algorithm for general costs. We use **_*δ*_** and **_*λ*_** to denote the cost of a duplication and a loss, respectively. We use **_*ρ*_**_0_ (respectively **_*τ*_**_0_) for the cost of an EGTcut (respectively EGTcopy) from the mitochondrial genome to the nuclear genome, and **_*ρ*_**_1_ (respectively **_*τ*_**_1_) for the cost of an EGTcut (respectively EGTcopy) from the nuclear genome to the mitochondrial genome. Note that the subscripts of the EGT costs indicate the source of the switch. Also denot*e*

$$\rho_{0}^{*}=\min(\rho_{0},\tau_{0}+\lambda )\;\;\rho_{1}^{*}=\min(\rho_{1},\tau_{1}+\lambda )$$

Roughly speaking, $\rho_{0}^{*}$ represents the minimum cost required to switch from mitochondrial to nuclear genome inside a branch of *T*, and $\rho_{1}^{*}$ the minimum cost required in the other direction. The purpose of $\rho_{0}^{*}$ and $\rho_{1}^{*}$ is that a switch can be accomplished by an EGTcut event, but also by an EGTcopy event followed by a loss.

Let x∈V(T). Note that $\tilde{s}(x)$ does not need to be inferred, since by Lemma 1, we can assume that $\tilde{s}(x)=lca_{S}(s(\ell (T[x])))$. Our dynamic programming table only needs to store the optimal cost on T[x] for each possible $\tilde{b}(x)\in \{0,1\}$. This requires testing each of three possible events $\tilde{e}(x)$ at *x*, and the number of scenarios to consider at *x* is therefore constant [this is the main reason for the gain in time compared to the algorithm of Doyon *et al.* (2010), which requires adding a dimension to the table corresponding to all possible species at *x*]. Let $b_{x}\in \{0,1\}$. We denote by $D[x,b_{x}]$ the minimum cost of a *DLE*-Reconciliation $\langle R,\tilde{s},\tilde{b},\tilde{e}\rangle$ of T[x] with *S* in which $\tilde{b}(x)=b_{x}$ (or ∞ if no such reconciliation exists). Trivially, if *x* is a leaf of *T*, we hav*e*

$$D[x,b_{x}]=\{\begin{array}{cc} 0 & \text{if}\;b_{x}=b(x)\\ \infty & \text{otherwise} \end{array}$$

Assume now that *x* is an internal node of *T*. Let *x_l_*, *x_r_* be the children of *x*. For $s_{1},s_{2}\in V(S)$, let $\mathrm{path}(s_{1},s_{2})$ denote the number of vertices on the path between *s*_1_ and *s*_2_ in *S*, *including s*_1_ and *s*_2_. Then defin*e*

$$l_{x}=\mathrm{path}(\tilde{s}(x),\tilde{s}(x_{l}))+\mathrm{path}(\tilde{s}(x),\tilde{s}(x_{r}))$$

which counts the number of mandatory losses on the child branches of a node *x* of *T*.

To compute $D[x,b_{x}]$, we use three auxiliary values $D[x,b_{x},e_{x}]$, where $e_{x}\in \{\mathrm{Spe},\mathrm{Dup},\mathrm{EGTcopy}\}$ represents the event label of *x* (note that *e_x_* cannot be an EGTcut event, since *x* has two children).

If $\tilde{s}(x)=\tilde{s}(x_{l})$ or $\tilde{s}(x)=\tilde{s}(x_{r})$, then $D[x,b_{x},Spe]=\infty$. Assuming this check has been performed, we hav*e*

$$\begin{array}{c} D[x,b_{x},Spe]=\lambda (l_{x}-4)+\sum_{x\prime \in \{x_{l},x_{r}\}}\min(D[x\prime ,b_{x}],\rho_{b_{x}}^{*}+D[x\prime ,1-b_{x}])\\ D[x,b_{x},Dup]=\delta +\lambda (l_{x}-2)+\sum_{x\prime \in \{x_{l},x_{r}\}}\min(D[x\prime ,b_{x}],\rho_{b_{x}}^{*}+D[x\prime ,1-b_{x}])\\ \;D[x,b_{x},\text{EGTcopy}]=\tau_{b_{x}}+\lambda (l_{x}-2)+\min\{\begin{array}{c} D[x_{l},b_{x}]+D[x_{r},1-b_{x}]\\ D[x_{l},1-b_{x}]+D[x_{r},b_{x}]\\ \rho_{1-b_{x}}^{*}+D[x_{l},b_{x}]+D[x_{r},b_{x}]\\ \rho_{b_{x}}^{*}+D[x_{l},1-b_{x}]+D[x_{r},1-b_{x}] \end{array} \end{array}$$

Put $D[x,b_{x}]=\min(D[x,b_{x},\mathrm{Spe}],D[x,b_{x},\mathrm{Dup}],D[x,b_{x},\mathrm{EGTcopy}])$. The value of interest is min(D[r(T),0],D[r(T),1]).

Theorem 1. *For any* x∈V(T)*and* $b_{x}\in \{0,1\}$*, the value of* $D[x,b_{x}]$*, as defined above, is equal to the minimum cost of a DLE-Reconciliation* $\langle R,\tilde{s},\tilde{b},\tilde{e}\rangle$*of* T[x]*with S satisfying* $\tilde{b}(x)=b_{x}$.

*Moreover, the minimum cost* min(D[r(T),0],D[r(T),1])*of a reconciliation of T with S can be computed in time* O(|V(T)|+|V(S)|).

Let us note that once the *D* table is computed, a standard backtracking procedure allow to reconstruct every optimal DLE-Reconciliation.

## 5 Experimental results

We implemented the above dynamic programming procedure in python in a software called EndoRex, which supports arbitrary costs as input and returns a reconciled gene tree in Newick format. The python source can be accessed at https://github.com/AEVO-lab/EndoRex. We then performed a variety of experiments on a dataset obtained from (Kannan *et al.*, 2014), as described bellow.

### 5.1 Kannan *et al.* (2014) dataset

For the reconstruction of evolutionary histories with EGT events, we used a dataset from Kannan *et al.* (2014) available at ftp://ftp.ncbi.nih.gov/pub/koonin/MitoCOGs. The dataset consists of 140 MitoCOGs extended with paralogs and nuclear protein-coding homologs from 2486 eukaryotes with complete mitochondrial genomes. MitoCOGs are clusters of orthologous genes for mitochondrial-encoded proteins generated using COG construction (Makarova et al., 2007; Yutin et al., 2009). Full description of the MitoCOG generation procedure is described in Kannan *et al.* (2014). Among the 140 MitoCOGs, 73 correspond to protein-coding gene families, 49 are hypothetical proteins and 18 are clusters for which the protein function is identified but not the gene name. Among these 73 MitoCOGs, 13 are core-mitochondrial proteins that are shared by most of the 2486 mitochondrial genomes. Statistics on MitoCOGs of the Kannan *et al.* dataset are given in Table 1.

**Table 1.** Statistics on the Kannan *et al.* (2014) dataset

Gene set	Nb of MitoCOGs	Nb of species	Nb of genes
Mitochondrial-encoded	140	2486	34 755
Nuclear-encoded	45	52	1317
Whole set	140	2486	36 072

*Note*: Notice that MitoCOGs have been designed for mitochondrial-encoded genes, and nuclear-encoded genes have been included later. This explains why all nuclear-encoded MitoCOGs, and the corresponding species, are included in the mitochondrial-encoded sets of MitoCOGs and species.

### 5.2 Dataset preprocessing

Among the 140 MitoCOGs of the initial Kannan *et al.* dataset, we first selected the 45 clusters involving nuclear-encoded protein sequences. Within these MitoCOGs, 52 eukaryotes are represented including 28 *Opisthokonta* (10 *Fungi*, 17 *Metazoa* and 1 *Choanoflagellata*), 9 *Viridiplantae*, 1 *Rhodophyta*, 1 *Glaucophyta*, 5 *Alveolata*, 1 *Amoebozoa*, 2 *Euglenozoa*, 1 *Heterolobosea*, 1 *Rhizaria* and 3 *Stramenopiles*. Based on Figure 1 in Kannan *et al.* (2014) and the analysis of the dataset, for the EGT evolutionary history inference with EndoRex, we selected the 11 plant species, including the 9 *Viridiplantae*, *Cyanidioschyzon merolae* (*Rhodophyta*) and *Cyanophora paradoxa* (*Glaucophyta*), as gene-content location is more diversified among this species group.

The 11 plant species are represented in 68 MitoCOGs with mitochondrial-encoded proteins and 41 MitoCOGs with nuclear-encoded proteins. We selected the clusters for which there were mitochondrial and nuclear encoded genes, yielding 28 MitoCOGS containing 326 protein-coding genes, including 184 encoded in the mitochondria and 142 in the nucleus. All the 28 MitoCOGs correspond to gene names that are present in the mitochondrial gene content review of Sloan *et al.* (2018).

Table 2 gives information about the 28 MitoCOGs of the 11 plants dataset specifying the gene name, the protein metabolic pathway and the number of genes and species for each MitoCOG.

**Table 2.** Statistics on the 28 MitoCOGs of the 11 plants dataset

MitoCOG	Gene	Metabolic	Nb of genes	Nb of
ID	name	pathway	(mito+nuc)	species
MitoCOG0006	nad3	Complex I	11 (10 + 1)	11
MitoCOG0007	nad4L	Complex I	13 (12 + 1)	11
MitoCOG0031	nad7	Complex I	11 (9 + 2)	11
MitoCOG0043	nad9	Complex I	11 (9 + 2)	11
MitoCOG0029	nad10	Complex I	13 (1 + 12)	10
MitoCOG0052	sdh2	Complex II	22 (1 + 21)	10
MitoCOG0051	sdh3	Complex II	8 (3 + 5)	6
MitoCOG0075	sdh4	Complex II	9 (4 + 5)	9
MitoCOG0003	cox2	Complex IV	13 (10 + 3)	11
MitoCOG0005	cox3	Complex IV	13 (10 + 3)	11
MitoCOG0059	atp1	Complex V	9 (7 + 2)	8
MitoCOG0076	atp4	Complex V	12 (11 + 1)	10
MitoCOG0004	atp6	Complex V	13 (12 + 1)	11
MitoCOG0014	atp9	Complex V	13 (10 + 3)	11
MitoCOG0027	rpl2	Translation	14 (5 + 9)	10
MitoCOG0053	rpl6	Translation	10 (4 + 6)	8
MitoCOG0092	rpl10	Translation	5 (2 + 3)	5
MitoCOG0048	rpl14	Translation	15 (5 + 10)	11
MitoCOG0039	rpl16	Translation	12 (8 + 4)	11
MitoCOG0070	rpl20	Translation	11 (2 + 9)	8
MitoCOG0080	rps2	Translation	9 (5 + 4)	9
MitoCOG0067	rps4	Translation	8 (7 + 1)	7
MitoCOG0061	rps7	Translation	12 (8 + 4)	11
MitoCOG0072	rps10	Translation	12 (3 + 9)	8
MitoCOG0054	rps11	Translation	12 (6 + 6)	10
MitoCOG0064	rps13	Translation	10 (7 + 3)	10
MitoCOG0055	rps14	Translation	9 (5 + 4)	8
MitoCOG0026	rps19	Translation	16 (8 + 8)	8

*Note*: For the ‘Nb of gene’ column, the number of mitochondria-encoded (mito) and nucleus-encoded (nuc) gene are specified.

**Fig. 3.**
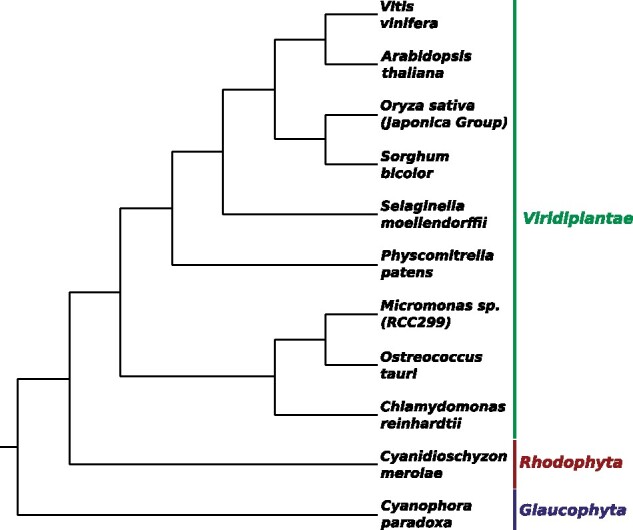
Species tree of the 11 plants considered in our experimental analysis. Topology of the tree is based on (Kannan *et al.*, 2014)

For each MitoCOG, we applied a pipeline to infer the evolutionary history of EGTs with DLE-Reconciliation along the 11 plants species tree. The topology of the species tree was taken from Kannan *et al.* (2014). We added the species *Micromonas sp. RCC299* as the sister species of *Ostreococcus tauri* as only these 2 among the 11 plants species belong to the *Mamiellophyceae* class. We also swapped the position between *P. patens* and *S. moellendorffi* according to (Puttick et al., 2018) (Fig. 3).

As for constructing gene trees, the first step of the pipeline was to align the protein sequences with MUSCLE (Edgar, 2004). In the second step, a maximum likelihood protein tree was infered using RAxML (v8.2.4) with the PROTGAMMAGTRX evolutionary model (Stamatakis *et al.*, 2014). NOTUNG (v.2.9.1.5) was then used to root the trees by minimizing the cost of a duplication-loss reconciliation with default parameter (loss cost: 1.0 and duplication cost: 1.5) (Stolzer et al., 2012).

The rooted protein trees obtained with this pipeline and the 11 plants species tree were given as input of the EndoRex software to infer a most parsimonious DLE-Reconciliation allowing for arbitrary costs for duplications, losses and EGTs.

### 5.3 EndoRex evolutionary events cost setting

As a reminder, we consider six parameters corresponding to the different evolutionary event costs: **_*δ*_** and **_*λ*_** the cost of, respectively, a gene duplication and loss; **_*ρ*_**_0_ (respectively **_*τ*_**_0_) the cost of an EGTcut (respectively EGTcopy) from the mitochondrial genome to the nuclear genome, and **_*ρ*_**_1_ (respectively **_*τ*_**_1_) the cost of an EGTcut (respectively EGTcopy) from the nuclear genome to the mitochondrial genome.

We test five different cost settings for the application of EndoRex on the 11 plants dataset. The setting *S*1 corresponds to the default values for parameters, with a unitary cost for evolutionary events (allowing to compute the DLE-Distance). For setting *S*2, the gene loss and duplication costs are those used in NOTUNG for rooting the protein trees, and EGTcopy and EGTcut costs are set higher to reflect the fact that these evolutionary events are less frequent than gene duplications: λ=1.0, δ=1.5 and $\rho_{0}=\rho_{1}=\tau_{0}=\tau_{1}=2.0$. In setting *S*3, we consider EGTcopy as less likely than EGTcut: $\lambda =1.0,\;\delta =1.5,\;\rho_{0}=\rho_{1}=2.0$ and $\tau_{0}=\tau_{1}=3.0$. For setting *S*4, we differentiate the cost of the mitochondria to the nucleus from the nucleus to the mitochondria gene move, and account for the fact that, during the evolution of eukaryotes, mitochondrial genes are integrated into the nuclear genome, while the reverse is extremely rare: $\lambda =1.0,\;\delta =1.5,\;\rho_{0}=2.0,\;\rho_{1}=3.0,\;\tau_{0}=3.0$ and $\tau_{1}=4.0$. Finally, setting *S*5 is the same as setting *S*4 except we make no difference between the costs of EGTcopy and EGTcut events: $\lambda =1.0,\;\delta =1.5,\;\rho_{0}=2.0,\;\rho_{1}=3.0,\;\tau_{0}=2.0$ and $\tau_{1}=3.0$.

Applied to the 28 MitoCOGs trees, EndoRex infers the same DLE-Reconciliation with the five different settings for 21 of the 28 MitoCOGs.

**Fig. 4.**
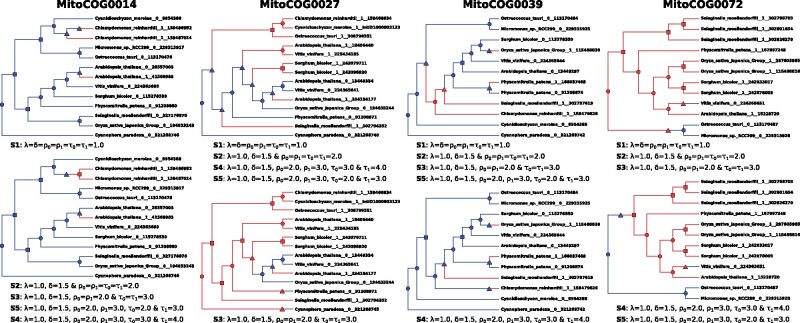
DLE-Reconciliations obtained for MitoCOG0014, MitoCOG0027, MitoCOG0039 and MitoCOG0072 with the EndoRex scores settings *S*1, *S*2, *S*3, *S*4 and *S*5. The blue part of the tree indicates that the genetic material is located in the mitochondrion, while the red part indicates location in the nucleus. The shape of an internal node represents its associated event, as represented in Figure 1 (circle for a speciation, rectangle for a duplication and triangle for an EGT event). Loss events are not represented. Genes are formatted as follow: [species name]__[gene-encoding location]__[gene id]. Moreover, 0 indicates a location in the mitochondrion, while 1 indicates a location in the nucleus

All the seven MitoCOGs with more that one inferred DLE-Reconciliation, depending on the considered setting, lead to two different DLE-Reconciliations: for MitoCOG0014, MitoCOG0051 and MitoCOG0053, setting *S*1 gives a DEL-reconciliation different from the other settings; for MitoCOG0027, it is setting *S*3 that gives a different DEL-reconciliation; for MitoCOG0005 and MitoCOG0039, it is setting *S*4; and finally for MitoCOG0072, the settings *S*4 ans *S*5 give a DEL-reconciliation different from *S*1, *S*2 and *S*3. We analyzed the two DLE-Reconciliations of MitoCOG0014 (*atp9*), MitoCOG0027 (*rpl2*), MitoCOG0039 (*rpl16*) and MitoCOG0072 (*rps10*) to illustrate the dynamic of the score settings (see Fig. 4).

**Fig. A1.**
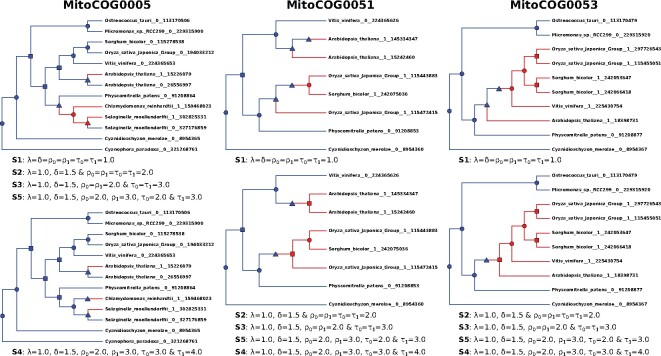
DLE-Reconciliations obtained forMitoCOG0005, MitoCOG0051 and MitoCOG0053 with the EndoRex scores settings *S*1, *S*2, *S*3, *S*4 and *S*5. The blue part of the tree indicates that the genetic material is located in the mitochondrion, while the red part indicates location in the nucleus. The shape of an internal node represents its associated event, as represented in Figure 1 (circle for a speciation, rectangle for a duplication and triangle for an EGT event). Loss events are not represented. Genes are formatted as follow: [species name]__[gene-encoding location]__[gene id]. Moreover, 0 indicates a location in the mitochondrion, while 1 indicates a location in the nucleus

According to these case studies, it seems that setting *S*1 is inappropriate as it leads to the prediction of higher number of EGTs which are rare evolutionary events (see MitoCOG0014 in Fig. 4, and MitoCOGs 51 and 53 in Appendix Fig. A1). For MitoCOG0027, setting *S*3 leads to the prediction of numerous EGTs from the nucleus to the mitochondria, which is very unrealistic as a very few number of gene movements from the nucleus to the mitochondria have been described in the literature. DLE-Reconciliations predicted with setting *S*4 are the scenarii most in line with the literature as it only infers EGTs from the mitochondria to the nucleus (except for MitoCOG0072), with transpositions located close to the leaves of the tree, indicating an ongoing process of endosymbiotic gene transfer in plants for this gene family (see MitoCOGs 39 and 72 in Fig. 4, and MitoCOG0005 in Appendix Fig. A1).

## 6 Conclusion

Investigating the origin, evolution and characteristics of gene coding capacity of eukaryotes has been among the central themes in the Life Sciences. In this context, the endosymbiotic origin of mitochondrial genomes and the gradual integration of the mitochondrial gene content to the nucleus are important evolutionary parameters expected to shed light on features of eukaryotic gene evolution and function.

From a computational point of view, detecting the footprint of endosymbiosis in the gene repertoires of the mitochondrial and nuclear genomes of eukaryotes requires new evolutionary prediction methods. This article is a first effort toward developing the appropriate algorithmic tools for analyzing the movement of genes inside a gene family between the mitochondrial and nuclear genome of the same species. We presented a linear-time algorithm computing a most parsimonious history of Duplication, Loss and EGT (DLE) events explaining a gene tree with leaves identified as mitochondrial or nuclear genes. We also presented a general dynamic programming algorithm, implemented in the EndoRex software, to compute all optimal DLE-Reconciliations for any arbitrary cost scheme of operations.

By applying EndoRex to a plant dataset, we showed that it is well-designed to infer the evolutionary histories of EGT events, considering a variety of cost settings. Some reconciled trees (not shown) of the 11 plants dataset produced evolutionary histories that could be considered unrealistic as leading to an unexpected high number of gene duplications and losses. As our algorithm is exact and thus guaranteed to infer the minimum number of events given a gene tree, this is likely due to errors in protein sequence alignment and/or gene tree inference, leading to erroneous gene trees (Hahn, 2007). A better gene tree inference pipeline should be designed in the future to get more accurate gene trees. In particular, gene trees have been rooted according to the DL-distance and standing on the default NOTUNG parameters. Instead, we could have rooted the trees according to our DLE-model, with the 5 considered cost settings. In addition, the obtained RAxML binary gene trees contain many weakly supported edges. Those edges may be contracted, and a polytomy resolution tool such as PolytomySolver (Lafond et al., 2016) may be used to better resolve multifurcations. On the other hand, simulations studies should also be conducted, in the future, to better evaluate the quality of the obtained solutions.

In fact, our method relies on a deterministic parsimony approach to compute all optimal DLE-reconciliations given a cost scheme for DLE events. This model has many limitations. In particular, parsimony does not allow to model multiple state changes along a branch of the phylogeny, or uncertainty in phylogenetic reconstructions. An alternative is to rely on approaches using stochastic state mapping models such as the mutational mapping approach (Bollback, 2006; Huelsenbeck et al., 2003). Since our method outputs all optimal DLE-reconciliations, it can also be used to compute the probabilities of all possible events over all optimal solutions.

Future algorithmic extensions of the optimization problem considered in this article may concern extending the model to account for both EGT and HGT events, toward inferring a Duplication, HGT, loss and EGT (DTLE) evolutionary scenario for a gene family. Another direction would be to infer common episodes of EGT events for a set of gene families. This may be handled by generalizing the Super-Reconciliation (Delabre et al., 2020) model to account for segmental DLE events.

Future developments will define an EGT simulation model to provide EGT evolutionary histories to assess the accuracy of our algorithm. Some efforts have been made to provide EGT simulation model. Brandvain and Wade (2009) provides a model to explore the influence of population-genetic parameters (such as selection, dominance, mutation rates and population size with a rate of self-fertilization) on the rate and probability of functional gene transfer from mitochondrial genome (haploid) to nuclear genome (diploid). (Kelly, 2020) defines an EGT simulation model based on the ATP biosynthesis cost for the encoding of a mitochondrial/chloroplast gene in the nuclear genome and the import of the resulting in the organelle. These prior works provide useful insights to design a model for the simulation of EGT evolutionary histories that would be strongly inspired from existing model for the simulation of HGT evolutionary histories.

Future applications will also concern a thorough analysis of protein-coding genes involved in common metabolic pathways. As an example, the oxydative phophorylation (OXPHOS) is a series of protein complexes (I, II, III, IV and V) leading to an electrochemical proton gradient activating the ATP synthase (complex V) that produces ATP. These protein-coding genes involved in OXPHOS are expected to share common mitochondrial-nuclear movements, as nucleus and mitochondria are two compartments with different biological dynamics.

Finally, the recent sequencing effort conducted toward jakobids and malawimonads protists genomes known to have emerged close to the eukaryotic origin will provide a valuable dataset that can be analyzed with the new developed algorithms, helping to shed light on a number of important biological questions, among them resolving the root of the eukaryote tree. In fact, as EGTs are rare events, candidate topologies for which DLE-Reconciliations infer the lowest number of EGT events, may provide evidence for a correct rooting.

*Financial Support*: Natural Sciences and Engineering Research Council of Canada;Fonds de recherche Nature et Technologie, Québec.

*Conflict of Interest*: none declared.

## Appendix A

Proof of Lemma 1 Let $\langle R,\tilde{s},\tilde{b},\tilde{e}\rangle$ be a DLE-Reconciliation of minimum cost between *T* and *S*. Let **_*λ*_** be the cost of a loss event. Let us first make an observation. Let v∈V(R) and let l∈ℓ(R[v])∩ℓ(T), assuming that *l* exists. Let $P=(v=p_{1},p_{2},\ldots ,p_{k}=l)$ be the path from *v* to *l* in *R*. It is easy to see from the definition of reconciliation that $\tilde{s}(v)=\tilde{s}(p_{1}),\tilde{s}(p_{2}),\ldots ,\tilde{s}(p_{k})=\tilde{s}(l)$ is a path of *S*, but with some vertices possibly being repeated (i.e. $\tilde{s}(p_{i})=\tilde{s}(p_{i+1})$ is possible, but otherwise $\tilde{s}(p_{i+1})$ is a child of $\tilde{s}(p_{i})$). It follows that $\tilde{s}(v)$ must be an ancestor of *s*(*l*). Since *v* and *l* were chosen arbitrarily, we have that for any $v\in V(R),\;\tilde{s}(v)$ is an ancestor of *s*(*l*) for every leaf l∈ℓ(R[v])∩ℓ(T). Now suppose that, for some $x\in V(R)\cap V(T),\;\tilde{s}(x)\neq lca_{S}(s(\ell (T[x])))$. Moreover, choose *x* as a lowest node of V(R)∩V(T) with this property (i.e. $\tilde{s}(x\prime )=lca_{S}(s(\ell (T[x\prime ])))$ for all descendants x′∈V(R)∩V(T) of *x* in *R*). Note that *x* is an internal node of *T* since $\tilde{s}(x)=s(x)$ for every leaf *x* of *T*. As we argued, $\tilde{s}(x)$ is an ancestor of *s*(*l*) for every leaf l∈ℓ(T[x]). Since $\tilde{s}(x)\neq lca_{S}(s(\ell (T[x])))$, it follows that $\tilde{s}(x)$ is a strict ancestor of $lca_{S}(s(\ell (T[x])))$. We first argue that *x* cannot be a speciation. Assume this is the case and let $x\prime_{l},\;x\prime_{r}$ be the children of *x* in *R* (but not necessarily in *T*). We use *x_l_* and *x_r_* to denote the children of *x* in *T*. By the definition of speciation, $\tilde{s}(x\prime_{l})$ and $\tilde{s}(x\prime_{r})$ are the two children of $\tilde{s}(x)$. Because $\tilde{s}(x)$ is a strict ancestor of $lca_{S}(s(\ell (T[x])))$, only one of $\tilde{s}(x\prime_{l})$ or $\tilde{s}(x\prime_{r})$ has descendants in {s(l):l∈ℓ(T[x])}. Assume without loss of generality that only $\tilde{s}(x\prime_{l})$ has such descendants. But then, $\tilde{s}(x\prime_{r})$ is not an ancestor of any member of s(ℓ(T[x])). In particular, $\tilde{s}(x\prime_{r})$ is not an ancestor of any member of $s(\ell (R[x\prime_{r}])\cap \ell (T))$, and the latter is easily seen to be non-empty (this is because $x\prime_{r}$ is an ancestor of *x_r_* and $T[x_{r}]$ has leaves from *T*). As we argued before, this is not possible, since there should be a path from $\tilde{s}(x\prime_{r})$ to any *s*(*l*) with $l\in \ell (T[x\prime_{r}])\cap \ell (T)$. Assume that *x* is a duplication or EGTcopy event (*x* cannot be an EGTcut event because it is binary). As before, let *x_l_* and *x_r_* be the children of *x* in *T* (but not necessarily in *R*). By the choice of *x*, $\tilde{s}(x_{l})=lca_{S}(s(\ell (T[x_{l}])))$ and $\tilde{s}(x_{r})=lca_{S}(s(\ell (T[x_{r}])))$. Thus $\tilde{s}(x)$ must be a strict ancestor of both $\tilde{s}(x_{l})$ and $\tilde{s}(x_{r})$. Let s′ be the child of $\tilde{s}(x)$ that is on the path from $\tilde{s}(x)$ to $lca_{S}(s(\ell (T[x])))$. We obtain an alternate reconciliation by modifying *R* to obtain another extension R′ of *T*. We do not change any event labeling. We map *x* to s′ and graft a loss in $\tilde{s}(x)$ on the edge between *x* and its parent in *R* (if any). In that manner, the parent of *x* in *R* still has a child mapped to $\tilde{s}(x)$ in R′. This increases the cost by **_*λ*_**, the cost of one loss. Now let $x_{1},x_{2},\ldots ,x_{k}$ be the nodes on the path from *x* to *x_l_* in *R* (excluding *x* and *x_l_*). Note that since *x* is a duplication or EGTcopy, $\tilde{s}(x)=\tilde{s}(x_{1})$. Moreover, at most one node among $x_{1},\ldots ,x_{k}$ can be an EGTcopy or an EGTcut, since there is no point in making more than one switch within an edge. If present, we may assume without loss of generality that such an event occurs at *x_k_*, the parent of *x_l_* in *R*, since the timing of the switch does not affect the reconciliation cost. In this case, $\tilde{s}(x_{k})=\tilde{s}(x_{l})=lca_{S}(s(\ell (T[x_{l}])))$. On the other hand, $\tilde{s}(x_{1})=\tilde{s}(x)\neq lca_{S}(s(\ell (T[x])))$. This implies that $x_{1}\neq x_{k}$, and thus *x*_1_ is not an EGTcopy or an EGTcut. It follows that *x*_1_ is a node inserted because of a grafted loss, and $\tilde{s}(x_{2})=s\prime$. In R′, we can remove *x*_1_ and its loss leaf, and by doing so, the left child of *x* becomes *x*_2_. This preserves all properties of a valid reconciliation because both *x* and *x*_2_ are mapped to s′. We can apply the same procedure on the path from *x* to *x_r_*. In R′, we have created one loss above *x*, but have removed two losses on both sides of *x*. No other event labeling has changed. Since we assume that losses have a non-zero cost, R′ has a strictly lower cost than *R*, a contradiction.

Proof of Lemma 2 We first show that the reconciliation $\langle R,\tilde{s},\tilde{b},\tilde{e}\rangle$ obtained from Algorithm 1 is a valid DLE-Reconciliation. Note that the tree *R* returned by the algorithm is the same as *R_DL_*, but with some grafted unary nodes for EGTcut events where needed. Consider some $x\in V(R_{DL})$. In *R*, we put $\tilde{e}(x)=\mathrm{Spe}$ if ${\tilde{e}}_{DL}(x)=\mathrm{Spe}$, and $\tilde{e}(x)\in \{\mathrm{Dup},\mathrm{EGTcopy}\}$ if ${\tilde{e}}_{DL}(x)=\mathrm{Dup}$. If no additional node was grafted as a new child of *x*, all properties of reconciliation would be preserved since we keep $\tilde{s}$ as in ${\tilde{s}}_{DL}$. If some node x′ was grafted as a new child of *x*, we ensure that $\tilde{s}(x\prime )$ is the same as the previous child of *x*, which ensures that we satisfy the properties of reconciliation. Therefore, we only need to check whether the tree *R_DL_* is modified in an appropriate way in the case of a different $\tilde{b}$ value for a node *x* of *T* and one of its two children *x_l_* or *x_r_*. Lines 2–8 first ensure that the starting tree *R* is such that, for each node *x* of *T*, $\tilde{b}(x)={\tilde{b}}_{T}(x)$, and for any edge (*x*, *y*) in *T* such that ${\tilde{b}}_{T}(x)\neq {\tilde{b}}_{T}(y)$, the corresponding path $\left(x,v_{1},v_{2},\ldots v_{n},y\right)$ on *R* is such that for all *i*, $\tilde{b}(v_{i})=\tilde{b}(y)$. Subsequently, in the case of a different $\tilde{b}$ value for a node *x* of *T* and its child *y*, the node *x* is either modified to an EGTcopy node, ensuring that the switch between $\tilde{b}(x)$ and $\tilde{b}(v_{1})$ is correctly explained by this EGTcopy, or a new EGTcut node *v* is grafted on the edge $\left(x,v_{1}\right)$, also correctly explaining the switch between $\tilde{b}(x)$ and $\tilde{b}(v_{1})$. We now show that the DLE-Reconciliation output by Algorithm 1 is of minimum cost. First Note that, from the initialization done in Line 8, for each leaf *x* which is on *R_DL_* but not in *T* (lost gene), the algorithm ensures that $\tilde{b}(x)=\tilde{b}(p_{x})$ were *p_x_* is *x’*s parent. Thus, grafted loss leaves never require an extra EGTcopy event on an ‘inserted edge’ of *R_DL_*. Assume another reconciliation $\langle R\prime ,\tilde{s}\prime ,\tilde{b}\prime ,\tilde{e}\prime \rangle$ has a strictly lower cost than $\langle R,\tilde{s},\tilde{b},\tilde{e}\rangle$ output by Algorithm 1. We first show that, for any node of *T*, the corresponding node in *R* and R′ have the same event label. Assume this is not the case. Let *x* be the lowest node of *T* such that $\tilde{e}\prime (x)\neq \tilde{e}(x)$. Let *x_l_* and *x_r_* be its two children in *T* and *v_l_* and *v_r_* be the two non-unary descendant of *x* in R′ the closest from *x*. Note that *x_l_* and *x_r_* do not necessarily correspond to *v_l_* and *v_r_* in R′. Rather, they may be strict descendants of these nodes in R′. 1. If ${\tilde{e}}_{DL}(x)=\mathrm{Dup}$, then from Algorithm 1, $\tilde{e}(x)=\mathrm{Dup}$ if $\tilde{b}(x_{l})=\tilde{b}(x)$ and $\tilde{b}(x_{r})=\tilde{b}(x)$, and $\tilde{e}(x)=\mathrm{EGTcopy}$ otherwise. As $\tilde{e}\prime (x)\neq \tilde{e}(x)$, we should have $\tilde{e}\prime (x)\in \{\mathrm{Spe},\mathrm{EGTcopy}\}$ in the first case, or $\tilde{e}\prime (x)\in \{\mathrm{Spe},\mathrm{Dup}\}$ in the second case.
Assume $\tilde{e}\prime (x)=\mathrm{Spe}$. From Lemma 1, as $\langle R\prime ,\tilde{s}\prime ,\tilde{b}\prime ,\tilde{e}\prime \rangle$ is a reconciliation of minimum cost, $\tilde{s}\prime (x)=lca_{S}(s(\ell (T[x])))$, and as *x* is a speciation node in R′, one of *v_l_* and *v_r_* should be mapped to $\tilde{s}{\left(x\right)}_{l}$ and the other to $\tilde{s}{\left(x\right)}_{r}$. Assume w.l.o.g. that $\tilde{s}\prime (v_{l})=\tilde{s}{\left(x\right)}_{l}$ and $\tilde{s}\prime (v_{r})=\tilde{s}{\left(x\right)}_{r}$. Now, as *x* is a duplication node in *R_DL_*, then $\tilde{s}(x_{l})=\tilde{s}(x)$ or $\tilde{s}(x_{r})=\tilde{s}(x)$. Assume w.l.o.g. that $\tilde{s}(x_{l})=\tilde{s}(x)$. As *x_l_* is a node of the subtree of R′ rooted at *v_l_*, by definition of a reconciliation, $\tilde{s}\prime (x_{l})$ should be a descendant of $\tilde{s}(v_{l})$, which is not the case as $\tilde{s}\prime (v_{l})=\tilde{s}{\left(x\right)}_{l}$ is rather a strict descendant of $\tilde{s}(x)=\tilde{s}(x_{l})=\tilde{s}\prime (x_{l})$. Therefore, *x* cannot be a speciation node in $\langle R\prime ,\tilde{s}\prime ,\tilde{b}\prime ,\tilde{e}\prime \rangle$. We deduce that $\tilde{e}\prime (x)\in \{\mathrm{Dup},\mathrm{EGTcopy}\}$. Now assume that $\tilde{b}(x_{l})\neq \tilde{b}(x)$ or $\tilde{b}(x_{r})\neq \tilde{b}(x)$. In this case, the algorithm puts $\tilde{e}(x)=\mathrm{EGTcopy}$ and, as *x* is not a speciation, it should be a duplication node in $\langle R\prime ,\tilde{s}\prime ,\tilde{b}\prime ,\tilde{e}\prime \rangle$. But then an a unary EGTcut node *v* should be present in one of the two paths from *x* to *x_l_* or from *x* to *x_r_* in R′, contradicting the fact that $\langle R\prime ,\tilde{s}\prime ,\tilde{b}\prime ,\tilde{e}\prime \rangle$ is a reconciliation of minimum cost, since labeling *x* as an EGTcopy node and removing *v* would reduce the cost of the reconciliation by one. Finally, assume that $\tilde{b}(x_{l})=\tilde{b}(x)$ and $\tilde{b}(x_{r})=\tilde{b}(x)$. In this case, the algorithm puts $\tilde{e}(x)=\mathrm{Dup}$ and, as *x* is not a speciation, it should be an EGTcopy node in $\langle R\prime ,\tilde{s}\prime ,\tilde{b}\prime ,\tilde{e}\prime \rangle$, which induces, by definition of an EGTcopy event, that one of the two children *y* of *x* in R′ is such that $\tilde{b}(y)\neq \tilde{b}(x)$. Now, as $\tilde{b}(x)=\tilde{b}(x_{l})=\tilde{b}(x_{r})$, one unary EGTcut node *v* should change the $\tilde{b}$ labeling of *y* to the $\tilde{b}$ labeling of its descendant in $\left\{x_{l},x_{r}\right\}$. But then relabeling *x* as a duplication node would allow removing *v* and thus reducing the cost of the reconciliation by one, contradicting the fact that $\langle R\prime ,\tilde{s}\prime ,\tilde{b}\prime ,\tilde{e}\prime \rangle$ is a reconciliation of minimum cost.
2. If ${\tilde{e}}_{DL}(x)=\mathrm{Spe}$, then from the properties of a DL-Reconciliation, we should have $\tilde{s}(x_{l})\neq \tilde{s}(x)$ and $\tilde{s}(x_{r})\neq \tilde{s}(x)$. From Algorithm 1, *x* remains a speciation node in $\langle R,\tilde{s},\tilde{b},\tilde{e}\rangle$.
As $\tilde{e}\prime (x)\neq \tilde{e}(x)$, we should have $\tilde{e}\prime (x)=\mathrm{Dup}$ or $\tilde{e}\prime (x)=\mathrm{EGTcopy}$. In both cases, $\tilde{s}(v_{l})=\tilde{s}(v_{r})=s(x)$. This implies that $x_{l}\neq v_{l}$ and $x_{r}\neq v_{r}$, and thus *v_l_* and *v_r_* are grafted because of losses. Since R′ uses the LCA-mapping by Lemma 1, we can remove *v_l_*, *v_r_* and their corresponding grafted loss leaves and make *x* a speciation, while preserving a valid reconciliation. This saves a cost of three (two losses and a *Dup* or *EGTcopy* event). In the worst case, we had $\tilde{e}\prime (x)=\mathrm{EGTcopy}$, in which case we can add an EGTcut event on the appropriate branch to enforce the same switch. Thus replacing the *Dup* or EGTcopy label of *x* by a speciation reduces the cost of R′ by at least two, contradicting the fact that R′ is a reconciliation of minimum cost.
Since we have the same number of *Dup* and ETTr events as R′, it remains to show that we cannot graft less nodes than those induced by Algorithm 1. The grafted nodes are either binary nodes corresponding to losses, or EGTcut unary nodes. Suppose R′ has less grafted nodes than *R*. Then there is an edge (*x*, *y*) in *T* such that the corresponding path $P\prime_{x,y}=(x,v\prime_{1},v\prime_{2},\ldots v\prime_{n\prime },y)$ in R′ is shorter than the corresponding path $P_{x,y}=(x,v_{1},v_{2},\ldots v_{n},y)$ in *R*. We consider a lowest edge (*x*, *y*) of *T* verifying this condition, and we assume, without loss of generality, that *y* = *x_l_*. Recall that by Lemma 1, $\tilde{s}(x)=\tilde{s}\prime (x)$ and $\tilde{s}(y)=\tilde{s}\prime (y)$.

- If ${\tilde{e}}_{DL}(x)=\mathrm{Dup}$, then *x* is a duplication or an EGTcopy node in both *R* and R′. Then, by definition of a reconciliation, $\tilde{s}(v_{1})=\tilde{s}(x)$. Moreover, from the fact that *R* is obtained from *R_DL_*, Algorithm 1 leads to a path $P_{x,y}$ with as many nodes as the path from $\tilde{s}(x)$ to $\tilde{s}(x_{l})$ in *S* if *x* is a duplication node, and an additional EGTcut node if $b_{T}(x)\neq b_{T}(x_{l})=b_{T}(x_{r})$. Moreover, it is easy to see that the number of losses crafted on (*x*, *y*) must be equal to the number of nodes on the path from $\tilde{s}(x)$ and $\tilde{s}(y)$, excluding $\tilde{s}(y)$, either in *R* or R′, and that the EGTcut event added by the algorithm cannot be avoided. And thus, the path $P\prime_{x,y}$ should be at least as long as $P_{x,y}$, contradicting the hypothesis that $P\prime_{x,y}$ is shorter than $P_{x,y}$.
- If ${\tilde{e}}_{DL}(x)=\mathrm{Spe}$, then *x* is a speciation node in both *R* and R′. Then, by definition of a reconciliation, $\tilde{s}(v_{1})=\tilde{s}\prime (v_{1})=\tilde{s}{\left(x\right)}_{l}$. Thus, from the fact that *R* is obtained from *R_DL_*, Algorithm 1 leads to a path $P_{v_{1},y}$ with as many nodes as the path from $\tilde{s}{\left(x\right)}_{l}$ to $\tilde{s}(x_{l})$ in *S*, with an additional EGTcut node if $\tilde{b}(x)\neq \tilde{b}(x_{l})$. Moreover, it is easy to see that no other operation (Spe, Dup, RGT or EGTcut) can allow making less losses or avoid the EGTcut event. And thus, the path $P\prime_{v_{1},y}$ should be at least as long as $P_{v_{1},y}$, contradicting the hypothesis that $P\prime_{x,y}$ is shorter than $P_{x,y}$.

Proof of Theorem 1 Let us first argue on the complexity of computing $D[x,b_{x}]$ for every x∈V(T) and every $b_{x}\in \{0,1\}$ (including D[r(T),0] and D[r(T),1]), our values of interest). The LCA-mapping $\tilde{s}$ can be computed in time O(|V(T)|+|V(S)|) using classical approaches from DL-reconciliation. We can compute D[x,0] and D[x,1] for every x∈V(T) in a post-order traversal of *T* (because their value only depends on *x_l_* and *x_r_*), and thus there are O(|V(T)|) values to compute. If we assume that if we have access to *l_x_* for each *x*, it is clear from the recurrences that $D[x,b_{x},\mathrm{Spe}],D[x,b_{x},\mathrm{Dup}]$ and $D[x,b_{x},\mathrm{EGTcopy}]$ can be computed in *O*(1) time. To access *l_x_* in time *O*(1) for any *x*, we can preprocess *S* by labeling each v∈V(S) by its depth (i.e. its distance to the root). Then, $\mathrm{path}(\tilde{s}(x),\tilde{s}(x_{l})$ is simply the difference in depth between $\tilde{s}(x)$ and $\tilde{s}(x_{l})$ (because $\tilde{s}(x_{l})$ must be a descendant of $\tilde{s}(x)$). This difference can be obtained in constant time, and it follows that *l_x_* can be obtained in *O*(1). Therefore, each $D[x,b_{x}]$ entry takes *O*(1) time to compute. Including the time to compute the preprocessing and the LCA-mapping, the total time of the algorithm is O(|V(T)|+|V(S)|). Let us now argue that the algorithm is correct. Let x∈V(T), let $b_{x}\in \{0,1\}$, and let $R=\langle R,\tilde{s},\tilde{b},\tilde{e}\rangle$ be a DLE-Reconciliation of minimum cost between T[x] and *S* that satisfies $\tilde{b}(x)=b_{x}$. The proof is by induction on the height of T[x]. If *x* is a leaf, it is easy to see that $D[x,b_{x}]$ is correct. Assume that *x* is an internal node with children *x_l_* and *x_r_*. We may inductively assume that $D[x_{l},b_{l}]$ and $D[x_{r},b_{r}]$ are computed correctly for $b_{l},b_{r}\in \{0,1\}$. In what follows, let $R_{l}=\langle R_{l},{\tilde{s}}_{l},{\tilde{b}}_{l},{\tilde{e}}_{l}\rangle$ be the reconciliation between $T[x_{l}]$ and *S* obtained by taking $R[x_{l}]$, and restricting $\tilde{s},\tilde{b}$ and $\tilde{e}$ to $V(R[x_{l}])$. Similarly, let $R_{r}$ be the reconciliation of $T[x_{r}]$ with *S* obtained by taking $R[x_{r}]$ and restricting $\tilde{s},\tilde{b}$ and $\tilde{e}$ to $R[x_{r}]$. We show two useful claims, the first being that these sub-reconciliations must be optimal with respect to their subtrees.

Claim 1.1. $c(R_{l})=D[x_{l},\tilde{b}(x_{l})]$ and $c(R_{r})=D[x_{r},\tilde{b}(x_{r})]$. Proof. By induction and by the definition of *D*, we have $D[x_{l},\tilde{b}(x_{l})]\leq c(R_{l})$. Moreover, in R we may replace the $R[x_{l}]$ subtree by $R_{l}$ (more precisely, replace $R[x_{l}]$ by *R_l_*, and use ${\tilde{s}}_{l},{\tilde{b}}_{l}$ and ${\tilde{e}}_{l}$ for the vertices of *R_l_*). Since ${\tilde{s}}_{l}(x_{l})=\tilde{s}(x_{l})$ and ${\tilde{b}}_{l}(x_{l})=\tilde{b}(x_{l})$, all conditions of a valid reconciliation are met after such a replacement. Furthermore, no additional loss, EGTcopy or EGTcut is required on the path between *x* to *x_l_*. If $D[x_{l},\tilde{b}(x_{l})]<c(R)$ held, this transformation would yield a lower cost reconciliation and contradict the optimality of R. Therefore, $D[x_{l},\tilde{b}(x_{l})]\geq c(R)$. It follows that $D[x_{l},\tilde{b}(x_{l})]=c(R_{l}])$. By a symmetric argument, $D[x_{r},\tilde{b}(x_{r})]=c(R)$. _**□**_

Claim 1.2. If $\tilde{e}(x)=\mathrm{Spe}$, then there are at least $l_{x}-4$ losses grafted on the $\left(x,x_{l}\right)$ and $\left(x,x_{r}\right)$ branches, and otherwise, there are at least $l_{x}-2$ such grafted losses. Proof. If $\tilde{e}(x)=\mathrm{Spe}$, in *R* there must be a loss grafted on the $\left(x,x_{l}\right)$ (respectively $\left(x,x_{r}\right)$) branch for each node of $\mathrm{path}(\tilde{s}(x),\tilde{s}(x_{l}))$ (respectively $\mathrm{path}(\tilde{s}(x),\tilde{s}(x_{r}))$), excluding $\tilde{s}(x)$ and $\tilde{s}(x_{l})$ (respectively $\tilde{s}(x_{r})$). The number of such losses is $l_{x}-4$ and induce a cost of $\lambda (l_{x}-4)$. If $\tilde{e}(x)\in \{\mathrm{Dup},\mathrm{EGTcopy}\}$, the required losses are the same, except that we do not exclude *x* from both paths, and thus $l_{x}-2$ losses are required for a cost of $\lambda (l_{x}-2)$. _**□**_

We now argue that $D[x,b_{x}]\leq c(R)$. First assume that $\tilde{e}(x)\in \{\mathrm{Spe},\mathrm{Dup}\}$. We then consider the four possible $\tilde{b}$ labelings of *x_l_* and *x_r_*.

• If $\tilde{b}(x)=\tilde{b}(x_{l})=\tilde{b}(x_{r})$, then no cost other than the losses is required on the $\left(x,x_{l}\right)$ and $\left(x,x_{r}\right)$ branches. Thus using claims 1.1 and 1.2,

$\begin{array}{c} c(R)\geq \{\begin{array}{cc} \lambda (l_{x}-4)+c(R_{l})+c(R_{r}) & \;\text{if}\;\tilde{e}(x)=\mathrm{Spe}\\ \delta +\lambda (l_{x}-2)+c(R_{l})+c(R_{r}) & \;\text{if}\;\tilde{e}(x)=\mathrm{Dup} \end{array} \end{array}$
$\begin{array}{c} =\{\begin{array}{cc} \lambda (l_{x}-4)+D[x_{l},b_{x}]+D[x_{r},b_{x}] & \;\text{if}\;\tilde{e}(x)=\mathrm{Spe}\\ \delta +\lambda (l_{x}-2)+D[x_{l},b_{x}]+D[x_{r},b_{x}] & \;\text{if}\;\tilde{e}(x)=\mathrm{Dup} \end{array} \end{array}$
Since for both $\tilde{e}(x)\in \{\mathrm{Spe},\mathrm{Dup}\},\;D[x,b_{x},\tilde{e}(x)]$ adds the losses, plus the minimum of $D[x\prime ,b_{x}]$ and $\rho_{b_{x}}^{*}+D[x\prime ,1-b_{x}]$ for each child $x\prime \in \{x_{l},x_{r}\}$, we see that $D[x,b_{x}]\leq D[x,b_{x},\tilde{e}(x)]\leq c(R)$.
• If $\tilde{b}(x)=\tilde{b}(x_{l})$ and $\tilde{b}(x)=1-\tilde{b}(x_{r})$, then no additional cost is required on the $\left(x,x_{l}\right)$ branch, but a switch is required on $\left(x,x_{r}\right)$. The minimum possible cost of such a switch is $\rho_{b_{x}}^{*}$, and thus using the two claims as the previous case (we omit the step replacing $c(R_{l})$ by $D[x_{l},b_{x}]$ and $c(R_{r})$ by $D[x_{r},1-b_{x}]$, which is implicit by claim 1.1), if $\tilde{e}(x)=\mathrm{Spe}$, we have
  $$c(R)\geq \lambda (l_{x}-4)+D[x_{l},b_{x}]+\rho_{b_{x}}^{*}+D[x_{r},1-b_{x}]$$

and if $\tilde{e}(x)=\mathrm{Dup}$, we have

$$c(R)\geq \delta +\lambda (l_{x}-2)+D[x_{l},b_{x}]+\rho_{b_{x}}^{*}+D[x_{r},1-b_{x}]$$

Again, the above expressions are considered by the minimization of $D[x,b_{x},\tilde{e}(x)]$, and so $D[x,b_{x}]\leq D[x,b_{x},\tilde{e}(x)]\leq c(R)$.

• If $\tilde{b}(x)=1-\tilde{b}(x_{l})$ and $\tilde{b}(x)=\tilde{b}(x_{r})$, this case is symmetric to the previous one.

• If $\tilde{b}(x)=1-\tilde{b}(x_{l})$ and If $\tilde{b}(x)=1-\tilde{b}(x_{r})$, then a switch with host *b_x_* is needed on both branches $\left(x,x_{l}\right)$ and $\left(x,x_{r}\right)$. Thus, if $\tilde{e}(x)=\mathrm{Spe}$, we have

$$c(R)\geq \lambda (l_{x}-4)+\rho_{b_{x}}^{*}+D[x_{l},1-b_{x}]+\rho_{b_{x}}^{*}+D[x_{r},1-b_{x}]$$

and if $\tilde{e}(x)=\mathrm{Dup}$, we hav*e*

$$c(R)\geq \delta +\lambda (l_{x}-2)+\rho_{b_{x}}^{*}+D[x_{l},1-b_{x}]+\rho_{b_{x}}^{*}+D[x_{r},1-b_{x}]$$

Again, these are considered in $D[x,b_{x},\tilde{e}(x)]$, and we get $D[x,b_{x}]\leq D[x,b_{x},\tilde{e}(x)]\leq c(R)$.

In all cases, $D[x,b_{x}]\leq c(R)$. It remains to show that this holds for $\tilde{e}(x)=\mathrm{EGTcopy}$. In this case, a cost of $\tau_{b_{x}}$ must be counted for the *x* node, plus the cost for $l_{x}-2$ losses by claim 1.2. Next, we consider all values of $\tilde{b}(x_{l})$ and $\tilde{b}(x_{r})$.

• if $\tilde{b}(x_{l})\neq \tilde{b}(x_{r})$, then as we argued

$$\begin{array}{c} c(R)\geq \tau_{b_{x}}+\lambda (l_{x}-2)+c(R_{l})+c(R_{r})\\ =\tau_{b_{x}}+\lambda (l_{x}-2)+D[x_{l},\tilde{b}(x_{l})]+D[x_{r},\tilde{b}(x_{r})] \end{array}$$

The latter expression is among the expressions that $D[x,b_{x},\mathrm{EGTcopy}]$ minimizes and thus $D[x,b_{x}]\leq D[x,b_{x},\mathrm{EGTcopy}]\leq c(R)$.

• if $b_{x}=\tilde{b}(x_{r})=\tilde{b}(x_{r})$, then since *x* is an EGTcopy event, one of the $\left(x,x_{l}\right)$ or $\left(x,x_{r}\right)$ branches must switch to $1-b_{x}$, then switch back to *b_x_*, implying a an EGTcut from $1-b_{x}$ to *b_x_* of cost $\rho_{1-b_{x}}^{*}$. In this situation,

$$c(R)\geq \tau_{b_{x}}+\lambda (l_{x}-2)+\rho_{1-b_{x}}^{*}+D[x_{l},b_{x}]+D[x_{r},b_{x}]$$

which is considered among the expressions minimized by $D[x,b_{x},\mathrm{EGTcopy}]$. Again, $D[x,b_{x}]\leq D[x,b_{x},\mathrm{EGTcopy}]\leq c(R)$.

• if $b_{x}=1-\tilde{b}(x_{l})=1-\tilde{b}(x_{r})$, then one of the $\left(x,x_{l}\right)$ or $\left(x,x_{r}\right)$ branches stays in *b_x_*, and thus must switch to $1-b_{x}$ for a cost of $\rho_{b_{x}}^{*}$. In this situation,

$$c(R)\geq \tau_{b_{x}}+\lambda (l_{x}-2)+\rho_{b_{x}}^{*}+D[x_{l},b_{x}]+D[x_{r},b_{x}]$$

which is considered among the expressions minimized by $D[x,b_{x},\mathrm{EGTcopy}]$. Again, $D[x,b_{x}]\leq D[x,b_{x},\mathrm{EGTcopy}]\leq c(R)$.

In every possible case, $D[x,b_{x}]\leq c(R)$.

We must now prove the complementary bound, i.e. that $D[x,b_{x}]\geq c(R)$. Let e∈{Spe,Dup,EGTcopy} such that $D[x,b_{x}]=D[x,b_{x},e]$. If *e* = *Spe*, the expression $D[x,b_{x},\mathrm{Spe}]$ corresponds to making *x* a speciation (which is possible since we check that neither of $\tilde{s}(x)=\tilde{s}(x_{l})$ nor $\tilde{s}(x)=\tilde{s}(x_{r})$ holds) and adding the minimum number of mandatory losses on $\left(x,x_{l}\right)$ and $\left(x,x_{r}\right)$. Let $b_{l}\in \{0,1\}$ that minimizes $\min(D[x_{l},b_{x}],\rho_{b_{x}}^{*}+D[x_{l},1-b_{x}])$, and define *b_r_* for *x_r_* analogously. Thus consider the reconciliation R′ in which *x* is a speciation, on which we graft the $l_{x}-4$ mandatory losses on $\left(x,x_{l}\right)$ and $\left(x,x_{r}\right)$ and then, for each of *b_l_* or *b_r_* that differs from *b_x_*, adds an EGTcut on the corresponding branch. Then, for $T[x_{l}]$ subtree, take an optimal reconciliation $R_{l}$ for $T[x_{l}]$ and for the $T[x_{r}]$ subtree, take the optimal reconciliation $R_{r}$ for $T[x_{r}]$. By induction, $R_{l}$ and $R_{r}$ are of costs $D[x_{l},b_{l}]$ and $D[x_{r},b_{r}]$ respectively. Since all optimal reconciliations use the LCA-mapping, such a reconciliation is valid and its cost is as defined in $D[x,b_{x},\mathrm{Spe}]$. It follows that $D[x,b_{x},\mathrm{Spe}]=c(R\prime )\geq c(R)$ (the latter inequality owing to the optimality of R).

If *e* = *Dup*, the argument is exactly the same, except that to construct R′, we make *x* a duplication and add $l_{x}-2$ losses instead.

Finally, assume that *e* = *EGTcopy*. It is not hard to see that each expression that $D[x,b_{x},\mathrm{EGTcopy}]$ may choose when minimizing corresponds to a valid reconciliation. Indeed, consider the reconciliation R′ where $\tilde{e}(x)=\mathrm{EGTcopy}$ for a cost of $\tau_{b_{x}}$. We add $l_{x}-2$ mandatory losses on the $\left(x,x_{l}\right)$ and $\left(x,x_{r}\right)$ branches. Then, the first two cases of the minimization in $D[x,b_{x},\mathrm{EGTcopy}]$ correspond to having no additional switch needed, and hence we can use the optimal reconciliation for $T[x_{l}]$ and $T[x_{r}]$. The third case corresponds to having both *x_l_* and *x_r_* mapped to *b_x_*, in which case we can choose to apply the EGTcopy on $\left(x,x_{l}\right)$, but need to switch back for a cost of $\rho_{1-b_{x}}^{*}$. The last case corresponds to having both *x_l_* and *x_r_* mapped to $1-b_{x}$, in which case the EGTcopy applies one switch, and we add an EGTcut for the other switch of cost $\rho_{b_{x}}^{*}$.

Since each possible case represents the cost of a valid reconciliation R′, we get $D[x,b_{x},\mathrm{EGTcopy}]=c(R\prime )\geq c(R)$. Thus for every possible value of *e*, we have $D[x,b_{x}]=D[x,b_{x},e]\geq c(R)$.

To conclude, the two complementary bounds show that $D[x,b_{x}]=c(R)$. _**□**_
